# The Responsibility for the Medical Care of Hospital Patients

**Published:** 1915-07-17

**Authors:** 


### THE RESPONSIBILITY FOR THE MEDICAL CARE OF HOSPITAL PATIENTS.

If this title is presented in the form of a question and a merely general answer is permitted, the problem is free from all difficulty. Clearly the responsibility for the care and treatment of patients admitted to hospital rests with the medical and surgical staff. But in any individual instance the decision becomes not a general but a particular one. Every patient, whether a hospital or a private patient, must necessarily be under the care of some individual practitioner, just as a ship must be under the charge and responsibility of some individual captain. Hence in a given instance it is not sufficient to say the staff is responsible. What is needed is the identification of the particular member of the staff with whom the duty lies. And when the question is thus driven home the answer may not be quite so evident as is above suggested.

The uncertainty, such as it is, in reference to the individual patient arises from the fact that each hospital patient has relations with at least two different practitioners. One of these is the resident officer, house physician or house surgeon as the case may be, and the other is the visiting physician or surgeon whose name is attached to the bed in which the patient lies. Thus the question here proposed resolves itself into this: Is it the resident officer or the visiting officer who, in relation to the individual patient, is the responsible practitioner in charge? Possibly it may be suggested that both are responsible, but this proposition cannot be accepted. There is no room for divided responsibility in the conduct of medical practice. Some one person is in charge of the sick man, and while he may ask for and receive assistance in counsel or in executive proceedings the final word and the consequences of this rest with himself. No doubt in private practice it is common to speak of a consultation with the object of "dividing responsibility." In such circumstances, supposing the practitioners concerned are in agreement, each of them does undoubtedly accept a special obligation or onus. But no practitioner has the duty of adopting the suggestions of a colleague unless his own judgment approves them, and the consultation once concluded, the decision of continuing or terminating the line of treatment agreed on rests with the acting medical adviser. Hence it is quite a fair thing to say that it is with this latter that the responsibility rests. It is he who exercises authority, and from this position obligation cannot be detached. A consultation with agreement merely means that the advice tendered is uttered with a stronger voice, while if there is disagreement the patient can only be directed to seek still further help. In either event some one person remains in charge of the patient and accepts the onus of the case. All this is a necessity of the situation, and the patient who is freed from it, and is the victim of various counsellors having no sure relations the one to the other, is profoundly to be pitied.

Now, whatever else may be said of the relation of the resident hospital officer to his corresponding visiting officer, it cannot be held that the latter acts only in a consulting capacity. Were this true he would only attend the hospital when summoned by his resident, whereas, as a matter of fact, he attends on stated occasions under the compulsion of the rules of the institution. Further, when he attends he is under no obligation to take the opinion of his resident or to discuss with the latter the course he proposes to follow. Nor, on the other hand, is the resident there to present his own views and advice, but rather to adopt and apply the directions which are given to him. If all this is true it is obvious that it is not the resident officer who is in charge of the case. His duty is not to exercise an independent therapeutic judgment, but to carry out the reasonable and legitimate orders of his more experienced senior. But as someone must be in charge, and as this cannot be the resident officer, there remains only the visiting member of the staff. Hence, applying the question with which we started, it is evident that each visiting physician or surgeon is the responsible practitioner for each of the patients admitted to hospital under his nominal care. In other words, his care of each of these patients is not merely nominal. On the contrary, his responsibility for the adequate care of each patient is complete. From this conclusion it follows that the duty of a visiting physician or surgeon cannot be ruled solely by the formal demands of the hospital rules. These may prescribe that he must visit the hospital perhaps daily or two or three times a week, as the case may be. But this cannot be the measure of his duty, for he is in charge of patients, and no hospital rules can say what amount of attention or frequency of attendance a sick man may require. The demands, of course, vary with the individual case, but whatever they are they have to be met; and to quote the satisfaction of hospital rules in answer to a suggestion of professional carelessness or negligence is to mistake the real nature and gravity of the charge. The bond of obligation is provided not by the letter of the law but by professional responsibility and by the patient's needs.

What, then, is the *rôle* of the resident officer? He is not, as is here shown, in charge of the patient, but has the duty of carrying out the directions of his senior. Further, it rests with him, being always at hand, to meet any sudden emergency which may arise, and to relate this in due course to the visiting practitioner in charge. If he proves himself to be incompetent or careless this does not remove the responsibility of the physician or surgeon in whose care the patient is placed. The duty of this latter is to provide effective treatment, and, if this is offered partly by means of a substitute, it is his responsibility to secure a substitute who is able and willing to do what he is told. Like a member of the Government, he must either censure his subordinate officer or support his actions. With a capable and industrious resident the visiting officer feels, no doubt, more confidence, but it is for him to satisfy himself by personal observation that this confidence is justified, for, whatever happens, he himself cannot escape responsibility. It is sometimes said in reference to the time occupied by hospital duties that members of the visiting staff have to attend only, say, twice a week. But no hospital physician or surgeon can limit his duty by a technical requirement, seeing that his true obligation is the welfare of the patients committed to his charge. It is under this expectation of highly experienced advice that patients seek admission to our hospitals and that a generous public finds the funds necessary for their administration. Hence a visiting officer who does not effectively take charge of his patients, but leaves them in charge of a junior, is denying the professions of the institution as well as failing in his individual obligations.

